# 9q22 Deletion - First Familial Case

**DOI:** 10.1186/1750-1172-6-45

**Published:** 2011-06-22

**Authors:** Linda Siggberg, Maarit Peippo, Marjatta Sipponen, Taina Miikkulainen, Keiko Shimojima, Toshiyuki Yamamoto, Jaakko Ignatius, Sakari Knuutila

**Affiliations:** 1 Department of Pathology, Haartman Institute and HUSLAB, University of Helsinki and Helsinki University Central Hospital, Haartmaninkatu 3, 00014 Helsinki, Finland; 2 Department of Medical Genetics, The Family Federation of Finland, Helsinki, Finland; 3 Institute for Integrated Medical Sciences, Tokyo Women's Medical University, Tokyo, Japan; 4 Department of Medical Genetics, Turku University Hospital, Turku, Finland

## Abstract

**Background:**

Only 29 cases of constitutional 9q22 deletions have been published and all have been sporadic. Most associate with Gorlin syndrome or nevoid basal cell carcinoma syndrome (NBCCS, MIM #109400) due to haploinsufficiency of the *PTCH1 *gene (MIM *601309).

**Methods and Results:**

We report two mentally retarded female siblings and their cognitively normal father, all carrying a similar 5.3 Mb microdeletion at 9q22.2q22.32, detected by array CGH (244 K). The deletion does not involve the *PTCH1 *gene, but instead 30 other gene,s including the *ROR2 *gene (MIM *602337) which causing both brachydactyly type 1 (MIM #113000) and Robinow syndrome (MIM #268310), and the immunologically active *SYK *gene (MIM *600085). The deletion in the father was *de novo *and FISH analysis of blood lymphocytes did not suggest mosaicism. All three patients share similar mild dysmorphic features with downslanting palpebral fissures, narrow, high bridged nose with small nares, long, deeply grooved philtrum, ears with broad helix and uplifted lobuli, and small toenails. All have significant dysarthria and suffer from continuous middle ear and upper respiratory infections. The father also has a funnel chest and unilateral hypoplastic kidney but the daughters have no malformations.

**Conclusions:**

This is the first report of a familial constitutional 9q22 deletion and the first deletion studied by array-CGH which does not involve the *PTCH1 *gene. The phenotype and penetrance are variable and the deletion found in the cognitively normal normal father poses a challenge in genetic counseling.

## Background

The first constitutional deletion of 9q22 was published in 1978 by Turleau [1] and since then only 29 patients have been reported, including two terminated fetuses. All have been sporadic (Table 1). In recent years, array comparative genomic hybridization (array CGH) has enabled more detailed reports on the genetic basis of 9q22 deletions. Reported deletion sizes vary from 2.3 Mb to more than 18 Mb and no recurrent breakpoints have been observed (Table 1). While most reported deletions are sporadic, three balanced parental chromosomal rearrangements involving 9q have been detected and two of these have likely predisposed to the deletion in the descendants [2,3] while one deletion lies outside the parental rearrangement [4]. Parental origin of the deleted chromosome has been traced in eleven cases of which eight were paternal [2 (patient 3), 3 (patient 1), 5-8,] and three maternal [3 (patient 2), 9, 10] (Table 1). Characteristic to all these deletions are non-recurrent breakpoints leading to variable gene composition and an inconsistent phenotype. Most of them, however, span the *PTCH1 *gene (MIM *601309) and associate with Gorlin syndrome or nevoid basal cell carcinoma (NBCCS, MIM #109400) due to haploinsufficiency of *PTCH1*.

**Table 1:** Summary of clinical and molecular features of previously reported patients with constitutional 9q22 deletions and the present ones.

Reference	Patient's age and gender	Postnatal height	Postnatal OFC	CNS features	Malformations	Dysmorphic features	Clinical Gorlin syndrome	Method of detection	Locus of the deletion	Parental origin and/or Parental chromosomal rearrangement	Size of deletion	*PTCH1*	*ROR2*
[28]	1y11m male	+ 3,8SD	> p 90	global delay hypotonia no falx calcification	right hydronephrosis left multicystic kidney left hand preaxial polydactyly	hypertelorism frontal bossing epicanthi low nasal bridge low-set ears auricular pits long philtrum high palate short, webbed neck sacral dimple	with reservations to young age	aCGH (Agilent 180 chip) and FISH	9q22.3 *de novo*		2,44 Mb	deleted	nr

[14]	2 y 3 m male	> p97	p75-97	moderate MR, wide cranial sutures, open posterior fontanelle	submucous cleft palate, pectus excavatum	epicanthic folds, wide and short neck, low nuchal hairline, wide nasal bridge, low-set and posteriorly rotated ears, micrognathia, widely spaced nipples, small teeth, deep plantar grooves	with reservations to young age	aCGH at 1 Mb resolution	9q22.32q31.1 *de novo*		6,54-8,12 Mb	deleted	nr

[5]	3 y 9 m female	+2,2SD	+3,5SD	normal development, hypotonia, spasticity,	cleft lip-palate, pigmented cyst on shoulder (ectopic meninx) ASD	frontal bossing epicanthic folds, broad eyebrows, synophrys, c-a-l spot on legs and arm	yes palmar & plantar pits	aCGH (Agilent 105A chip)	9q22.32 *de novo*	paternal	2.3 Mb	deleted	nr

[26, patient 1]	12 y male	-1,1SD	+1,7SD	moderate MR, seizures/epilepsy		epicanthi mouth small upper lip thin strabismus	yes falx calcification, frontal ganglioglioma, rib anomalies, odontogenic ceratocysts, palmar and plantar pits	FISH with BAC clones	9q21.33q22.33 *de novo*		15,33-16,04 Mb	deleted	nr

[26, patient 2]	23 y male	+1,9SD	+2,6SD	severe MR, seizures, trigonocephaly, craniosynostosis,	cleft lip-palate retinal detachement, cataract, glaucoma double urethra	hypertelorism	yes odonto-genic ceratocysts, thyroid adenocarcinoma	FISH with BAC clones	9q21.33q31.1 *de novo*		18,08-18,54 Mb	deleted	nr

[27, restudy of patient 1 originally presented in 33]	50 y female	normal	macrocephaly	mild MR, hypotonia,	"rib and bone anomalies" kidney problems, one eye blind	frontal bossing, palpebral fissures slant down, epicanthi, maxillary prognatism,, dense eyebrows, dental anomalies, delayed dental eruption	yes basocellular carcinomas, jaw cysts, intracranial calcification, palmoplantar pits	quantitative multiplex fluorescent PCR, polymorphic markers, long-range PCR, sequencing	9q22.32q22.33 *de novo*		4,5 Mb	deleted	nr

[25, patient G10]	8 y male	nr	nr	severe MR, epilepsy, hydrocephalus	inguinal hernia, polydactyly scoliosis	hypertelorism	yes palmar & plantar pits	HR microarray	inv(9)(q21.2q33.1) = 9q22.32 92,934,973/92,945,040-98,137,216/98,141,889		5 Mb	deleted	nr

[16, patient G5, restudy of patient originally presented in 34]	12 y male	nr	>p97	severe MR, epilepsy, dilated lateral ventricles, thin corpus callosum, hydrocephalus	hydronephrosis, scoliosis	hypertelorism epicanthi webbed neck	yes basal cell carcinoma, palmar and plantar pits, multiple jaw cysts odonto-genic ceratocysts, calcification of falx and tentorium cerebelli	HR microarray	9q21.31q22.31 *de novo *88,656,506/88,656,835-99,686,554/99,687,352		11 Mb	deleted	deleted

[17]	12 y female	nr	normal	severe MR, brain atrophy	laryngeal stenosis, pulmonary valve stenosi, pectus excavatum, kyphoscoliosis, hypoplastic clavicles	down-slanting palpebral fissures, epicanthi, prognatism, asymmetric palpebral fissures, broad eyebrows, synophrys high forehead, pointed chin short neck, c-a-l-spots	yes	aCGH	9q22.1q22.32 *de novo*		7,7 Mb	deleted	deleted

[6]	13 y female	nr	nr	MR, ventriculomegaly	mild macroglossia	hypertelosim, frontal bossing, epicanthi, ears posteriorly rotated, teeth small, prominent gingivae, toenails hypoplastic, mild hemihypertrophy	yes mandibular cysts, plantar and palmar spots, rhabdomyosarcoma, Wilms tumor	karyotype,polymorphic markers at the *PTCH1 *region	9q22q32 *de novo*	paternal	nr	deleted	nr

[7, patient 1]	5 y male	+2,5SD	+2SD	severe MR	umbilical hernia, pectus excavatum, trigonocephaly- craniosynostosis	epicanthi mouth small upper lip thin ear pits ears low set ear lobules uplifted hyperlaxity, short neck,	with reservations to young age	aCGH at 1 Mb resolution	9q22.32q22.33 *de novo*	paternal	< 6,5 Mb	not tested	nr

[7, patient 2]	8 y female	+2SD	>+3SD	severe MR, seizures trigonocephaly ventriculomegaly thin corpus callosum	thyroglossal cyst with sternal fistula, no dentition, umbilical hernia, pectus excavatum, kyphosis	epicanthi, palpebral fissures slant down, small mouth, thin upper lip, thick ears indentation of ear lobules. short neck	with reservations to young age	aCGH at 1 Mb resolution	9q22.32q22.33 *de novo*	paternal	< 6,5 Mb	not tested	nr

[27]	5 m male	p75-90	p90-97	MR, hypotonia seizures hydfrocephalus caused by compression by cerebellar vermis	inguinal hernias, PDA, undescended testes, high arched palate, postaxial polydactyly of left foot, cervical ribs	hypertelorism, prognatism, broad face and forehead, broad nasal bridge, supraorbital ridges well developed	yes	HR karyotype, aCGH, FISH	9q22.32q31.3 *de novo*	paternal	12 Mb	deleted	nr

[15]	21 y male	+0,2SD	-1SD	mild MR	kyphosis, postaxial polydactyly, mild pulmonary valve stenosis, inguinal hernias, undescended testes, hypodontia of permanent teeth, palate high arched, uvula bifid, bilateral nasal stenosis, taurodonty of 2^nd ^molars	frontal bossing epicanthi, palperbral fissures slant down, prognatism, synophrys, hypotelorism, excess nuchal skin, ears low-set and posteriorly rotated, nares anteverted, lips thick, face high	yes calcification of cella turgica and falx cerebri, basal cell nevus carcinomas, jaw cysts	HR karyotype, FISH with BAC clones	9q21.3q31 *de novo*		15.3-15.6 MB	deleted	deleted

[32]	29 y female	short	nr		poor vision, telangiectatic nodule on skin hemivertebra T5, scoliosis, elongated clavicle	hypotelorism, ulnar deviation of hands	yes multiple basaliomas, calcification of falx, tentorium cerebri and cella turcica, mandibular cysts	karyotype	9q22.1q31.2	parents not available	nr	assumed deleted	nr

[4]	12 y female	nr	nr	mild MR, bridging of cella turcica, broad cavum septi pellucid, dilated cerebral ventricles	hyperopia, deverticulum of the renal calyx, occult spina bifida L5-S4, pectus excavatum, bilateral patellar dysplasia, unsual clavicles, exostosis of distal phalanx of thumb thumbs, abnormal hypoplasia of maxilla	hypertelorism, biparietal bossing, epicanthi, palpebral fissures slant down, prognatism, synophrys, webbed neck, synophrys and broad eyebrows, low midface, broad nose tip, low set, posteriorly rotated ears	yes basalioma basal cell nevi trichoepithelioma	HR karyotype, FISH	9q22.32q33.2 *de novo *outside the maternal translocation	familial t(9;17)(q34.1p11.2)mat	nr	deleted	nr

[30, patient A]	age nr, female	nr	nr	MR			nr	karyotype, aCGH	9q21q22.1		nr	nr	deleted

[30, patient B]	age nr, female	nr	nr	MR			nr	karyotype, aCGH	9q22.1q31.2		nr	nr	deleted

[8]	6 y male	p50	>p97	severe MR hypotony	PDA, severe scoliosis, fingers slender, 5^th ^finger camptodactyly, palate high arched, short metacarpals and distal phalanges	frontal bossing, epicanthi, palpebral fissures slant down, ears low-set, hypoplastic nostrils, micrognarhia, small nails	with reservations to young age increasing nr of nevi, sole pits	HR karyotype, FISH, genotyping	9q22.31q31.2 *de novo*	paternal	D9S303 ->D9S930	deleted	deleted

[3, patient 1]	15 y female	<p3	p75	MR, hydrocephalus with shunt, corpus callosum agenesis	inguinal hernias, bilateral conductive hearing loss, ectopic eruption of incisors, occult spina bifida T2-T3, scoliosis	frontal bossing, synophrys, prognathism	yes palmoplantar pits, bifid ribs	HR karyotype, RLFP polymorphisms	9q22q22 *de novo*	paternal	10-16 cM	deleleted	deleted

[3, patient 2]	26 y female	short	microcephaly	MR	PDA, CoA, anomalous right subclavian artery, bilat conductive hearing loss	frontal bossing, hypertelorism, prognatism, prominent supraorbital ridges, high palate	yes multiple basal cell carcinomas, leiomyoma coli, multiple bifid ribs odontogenic ceratocysts, ameloblastoma	HR karyotype, RLFP polymorphisms FISH	9q22q32 *de novo*	maternal t(ins[9][p22q32q22];16)(p22;q21) mat	22-39 cM	deleted	deleted

[9]	14 y male	<p3	<p3	severe MR brachycephaly, dilated ventricles	undescended testes, left pes equinovarus, I partial I-IV toe syndactyly,	frontal bossing, hyperteloris, epicanthi, palpebral fissures slant down, broad eyebrows, wide mouth, thick lips, irregular dentition, ears small, no ear lobes, short neck, wide internipple distance, hypoplastic genitalia, tapering fingers, small toes, medial deviation of toes	nr	karyotype	9q22q32 *de novo*	maternal	nr	nr	nr

[21]	15 m male	p25	p75	severe MR, partial aplasia of corpus callosum, dilatation of ventricles	laryngomalacia, cleft palate, PDA, abnormal aortic valve, epiglottic dysplasia, abnormal vocal cords	frontal bossing, palpebral fissures slant down, hypertrichosis, long eyelashes, epicanthus inversus, nose short, brodge depressed, nares upturned, long philtrum, small mouth, thin upper lip, receding chin, ears large and low set, large lobules, loose skin on cheeks	nr	karyotype (500 bands)	9q22q2207 *de novo*		nr	nr	nr

[2, patient 3]	7 m female	p90	p75	mild to moderate MR, brachycephaly, hydrocephalus	VSD, PDA hallux valgus	hypertelorism,epicanthi, synophrys, ptosis, philtrum short ears posteriorly rotated	nr	HR karyotype	9q22.3q31.1 *de novo*	paternal dir ins(4;9)(q33;q22.3q31.1) pat	nr	nr	nr

[35]	infant male	nr	nr	asymmetric ventricles, partial fusion of cerebellar hemispheres, polymicrogyria, delayed cerebellar neuronal migration, enlarged massa intermedia	cryptorchidism, focal glomerulosclerosis, accessory spleen, partial fusion of vertebrae D2 and D3, pectus excavatum, irregular ribs interphalangeal ankylosis		nr	karyotype	9q22q32 *de novo*		nr	nr	nr

[10]	3 m male	nr	nr	death at 3 months, seizures	duodenal atresia, malrotation, Meckel diverticulum, multilobulated spleen, accessory spleens renal dysplasia, hydroureter polydactyly of hand, syndactyly of feet angulated clavicles thorax asymmetric	palpebral fissures slant down, epicanthi, hypotelorism, hirsutism, short palpebral, fissures, depressed nasal bridge, auditory canals narrow, philtrum long	nr unusual ribs,	karyotype, Q, C and R bands	9q22q32 *de novo*	maternal	nr	nr	nr

[1, patient 1]	14 y male	-1SD	0SD	severe MR, epilepsy		hypertelorism, palpebral fissures slant down, nose short, nares anteverted, philtrum long, mild micrognathia	nr	karyotype with R bands	9q11q22 *de novo*		nr	nr	nr

Present case 3	37 y male	-1SD	-0,25SD	normal cognition, dysarthria	grade IV vesicoureteral reflux, hydroureter and hypoplastic left kidney. funnel chest, three lower-most costal cartilages broadly fused	deepset and small toenails, palpebral fissures slant down, high bridged nose, narrow nares, long deep furrowed philtrum, ears with broad helices and uplifted lobuli, short 2^nd ^finger nails	no	aCGH	9q22.2q22.31 de novo		5,3Mb	not deleted	deleted

Present case 1	8,5 y female	0,1SD	+0,5SD	moderate MR, dysarthria		short neck, slight ptosis on the right, downward slant of the palpebral fissures, narrow nose, small nares, long philtrum with a narrow deep groove, tented upper lip, ears with broad helices and uplifted lobuli. toe nails II-V bilaterally small	no	aCGH	9q22.2q22.31	paternal	5,3 Mb	not deleted	deleted

Present case 2	4 y female	-0,2SD	+1SD	moderate MR, dysarthria		down slanting palpebral fissures, mild left side ptosis, narrow nares, uplifted ear lobuli and thick helices, long philtrum with a deep furrow, thin and tented upper lip.	no	aCGH	9q22.2q22.31	paternal	5,3Mb	not deleted	deleted

All values given as reported in each original paper.

Abbreviations: nr = no report, y = year, m = month, OFC = head circumference; CNS = central nervous system; MR = mental retardation; p = percentile; SD = standard deviation, aCGH = array comparative genomic hybridization, HR = high resolution.

We describe a family where two mentally retarded siblings and their cognitively normal father share an identical 5,3 Mb deletion at 9q22.2q22.32, not including *PTCH1*, and discuss genotype-phenotype correlation in these patients.

## Methods

### Cytogenetic analysis

Chromosome metaphase spreads of patients 1, 3, and the healthy mother were analyzed by standard G-banding karyotype analysis (400 band resolution). Additional subtelomere-fluoresence *in situ *hybridization (FISH) analysis was conducted on patient 1 according to standard protocols. Targeted FISH-analysis of lymphocyte cells of patient 3 was performed using BAC-probe RP11-30L4.

### Molecular karyotyping

Mental retardation in addition to subtle but undisputable dysmorphic features in patients 1 and 2 were indications to perform additional high-resolution analysis by comparative genomic hybridization (CGH) and single nucleotide polymorphism (SNP) arrays. Standard molecular karyotyping was performed using a 244 K CGH array (Agilent Technologies, Santa Clara, CA, USA), as previously described [11].

Array CGH results were confirmed by SNP array analysis of patients 1 and 3 and the paternal grandparents, using the Genome-Wide Human SNP array 6.0 according to manufacturer instructions (Affymetrix, Santa Clara, CA, USA). The SNP 6.0 array contains 906,000 SNP probes and 946,000 copy number probes and has an average resolution of 0.7 kb. Data was extracted using the Genotyping console software V.3.0.2 with default settings, including the BRLMM-P-Plus algorithm and the Hidden-Markow Model for smoothing the copy number data. As a reference set we used data from 90 Caucasian HapMap samples. The extracted data was further analyzed using the Chromosome Analysis Suite software V.1.0. Copy number changes were called and filtered based on reference data and a minimum amount of 10 consecutive probes. All changes that were called were further compared to the Database of Genomic Variants (DGV, http://projects.tcag.ca/variation) as well as in-house data (unpublished material). All array data is stored in and available from the CanGEM Database (http://www.cangem.org). Array CGH and SNP data were analyzed using the reference genome build 18 (NCBI 36).

### Clinical report

**Figure 1:**
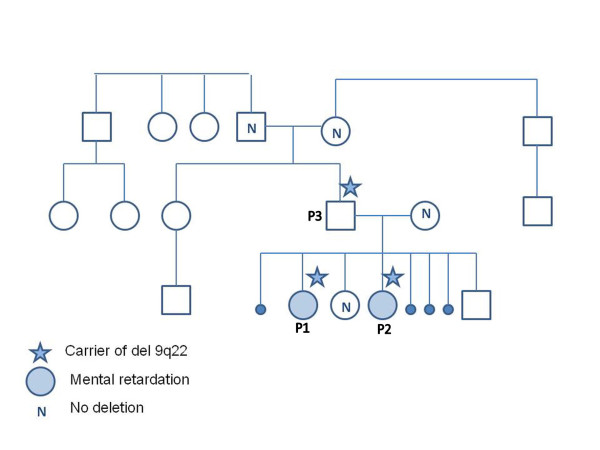
**Pedigree of the family **Patients 1, 2, and 3 all have the deletion of 9q22, as indicated by the star. The healthy mother, sister, and paternal grandparents, do not have the deletion, as indicated by N. The healthy brother was not tested for the deletion. Only patients 1 and 2 (P1, P2) have mental retardation.

The family has four children and in addition the mother has a history of four first trimester miscarriages. Patients 1 and 2 are the 2^nd ^and 3^rd ^born children in the family. The psychomotor development of the eldest daughter has been within normal limits, but she suffers from short attention span and need special support at school. The youngest boy is considered healthy at 10 months of age. The paternal grandparents have neither learning problems nor congenital abnormalities and the family history is unremarkable (Figure 1).

**Patient 1**, a girl, was born after an uneventful pregnancy. She had normal birth size (3450 g, 49 cm, OFC 35 cm) and Apgar score was 10 at 1 and 5 minutes. A drop in blood hemoglobin from 172 g/l to 127 g/l was observed during the first day and she received a red cell transfusion. No evidence for bleeding, infection, hemoglobin abnormality, or immunization were found. She was discharged in good condition at the age of one week. She has suffered from recurrent middle ear infections since 1 year of age and repeated insertion of grommets and prophylactic antibiotics were not helpful. She developed bronchial asthma at the age of four years. At 2 and 3 years she had febrile convulsions but EEG recordings were normal.

She learned to walk independently at 20 months and spoke her first words at 15 months. At 4 years neuropsychological examination showed mild mental retardation, defective linguistic development and clumsy fine motor performance. At 9 years she attends a school for developmentally handicapped children and has learned elements of reading and writing. She needs help in basic daily skills. She speaks using sentences, but her speech is dysarthric. The vicious circle of ear infections continues. She needs treatment for asthma and uses melatonin for sleep problems.

**Figure 2:**
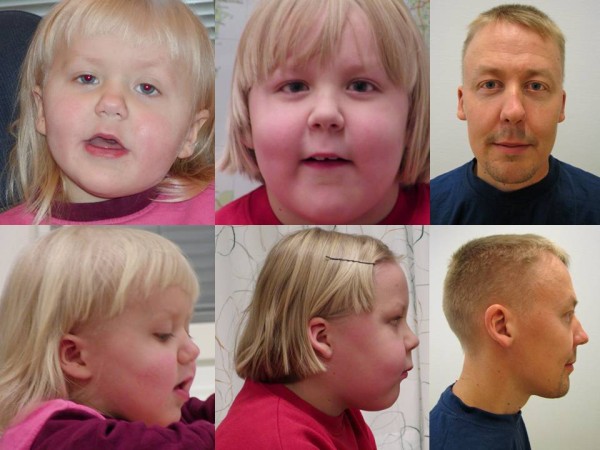
**Facial features of the patients **Facial features of patients 1 to 3 from left to right. Note short neck, slight unilateral ptosis, downward slant of the palpebral fissures, narrow nose, small nares, long philtrum with a narrow deep groove, tented upper lip, ears with broad helices and uplifted lobuli.

**Figure 3:**
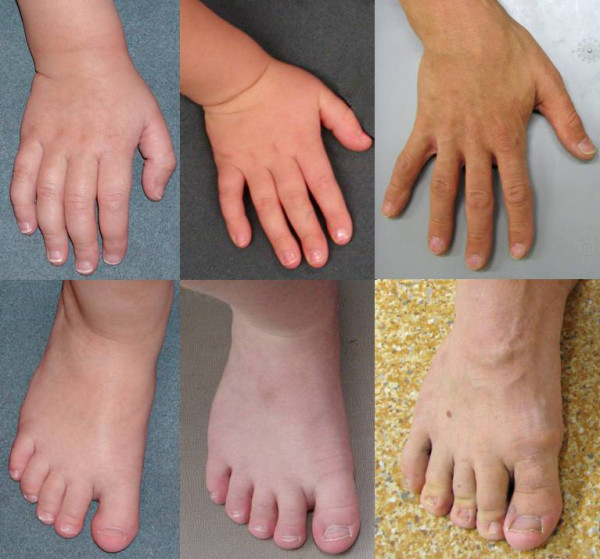
**Hands and feet of the patients **Hands and feet of patients 1 to 3 from left to right. Note that patient 1 has small toe nails II-V on both feet and patient 3 has small and deep-set toenails and his 2^nd ^finger nails are short.

During the first two years her height was at +2SD and has thereafter approached +0.5SD (target height -0.5SD). She has overweight (BMI 24) since 2 years of age. The OFC grows steadily at -0.5SD. She has normal body proportions, short neck, slight ptosis on the right, downward slant of the palpebral fissures, narrow nose, small nares, long philtrum with a narrow deep groove, tented upper lip, ears with broad helices and uplifted lobuli. Her toe nails II-V are small on both feet; otherwise feet, toes, hands, fingers and fingernails are normal (Figures 2 and 3).

Blood lymphocyte counts have repeatedly been normal. She has normal immunoglobulin levels including IgG subclasses, C3 and C4 and alpha-1-antitrypsin and no IgE related allergies have been found. Urine metabolic screen was negative an no vacuolated lymphocytes were detected. The brain MRI and US of kidneys and abdominal organs were normal at the age of 4.5 years. X-rays of the thorax showed no skeletal anomalies. Chromosome analysis (400 bands), subtelomere-FISH, 22q11 FISH (TUPLE1 probe), and FRAXA mutation analysis were normal.

**Patient 2 **is the younger sister of patient 1. She was born at term after an uneventful pregnancy. The mother was treated with acyclovir for herpes simplex type1 infection from the 17^th ^week up until the delivery and with hydroxysitsin for her itching. Her birth size was normal (3770 g, 52 cm, OFC 35 cm) At the age of three weeks she was admitted to hospital for apneas caused by pulmonary and middle ear infections and needed mechanical ventilation for four days. The causative microorganism was not identified. EEG recording showed right sided spikes and phenobarbital treatment was started. At 6 months the medication was discontinued due to absence of epileptic symptoms and normal EEG. Brain MRI, cardiac conduction examinations, and cardiac ultrasound were normal.

Psychomotor developmental delay was obvious since the age of 6 months. She learned to walk independently at 2.5 years, spoke words at 3 years and short sentences at 5 years of age. At 5 years her speech is dysarthric, she has problems of falling asleep in the evening and treatment with melatonin has shown modest success. She has sudden attacks of aggression and abrupt standstills when she is withdrawn from contact and stares or cries and holds her head between her hands. EEG recordings show no epileptic activity. Her ability of reciprocal contact is very defective. She also suffers from abundant ear infections and from a tendency to asthmatic bronchitis. During the 1^st ^year her height increased from +1SD to +2SD and then has declined to -0,5SD (her target height). Her OFC has grown at 0SD during the first 6 months of life and thereafter has remained at +1SD. She shows down slanting palpebral fissures, mild left side ptosis, narrow nares, uplifted ear lobuli and thick helices, long philtrum with a deep furrow, thin and tented upper lip (Figures 2 and 3). Her immunoglobulin levels are normal, but IgE related food allergy has been suspected.

**Patient 3 **is the father of patients 1 and 2. He was born term after a normal pregnancy. His birth size was normal (53 cm, 4090 g) and Apgar score was 9 at 1 and 10 at 5 minutes. At the age of 2.5 months he had a febrile pyelonephritis and urological examinations revealed grade IV vesicoureteral reflux, hydroureter and hypoplastic left kidney. In adult age his renal function has been normal. He suffered from recurrent middle ear infections and secretory otitis up to his teens. Surgery to correct his funnel chest was attempted when he was 6 years old. The surgery report describes the three lower-most costal cartilages being broadly fused. The immediate postoperative X-ray could be traced and it showed normal claviculae, ribs and thoracic vertebrae.

His psychomotor development during childhood was considered normal except speech development and he had speech therapy up to his teens. At adult age he still suffers from dysarthria. His school and military service history are unremarkable. Presently he works as a mailman and studies to become a nurse's aide.

At 1 year of age his height was 80 cm (+1SD). His adult height is 171,5 cm (-1SD), weight 68 kg (BMI 23) and OFC 57 cm (-0,8SD). He has partial upper denture after removal of decayed front teeth. The thoracic cage shows sequelae of childhood surgery and the sternum appears short but body-limb proportions are normal. He has down-slanting palpebral fissures, high nasal bridge, narrow nares, long philtrum with deep furrow, and the ears have broad helices and uplifted lobule. The 2nd finger nails are short and he has deep set toe nails. (Figures 2 and 3).

## Results

Karyotype analysis of patients 1, 3, and the mother, were normal. Sub-telomere FISH analysis of patient 1 was also normal.

**Figure 4:**
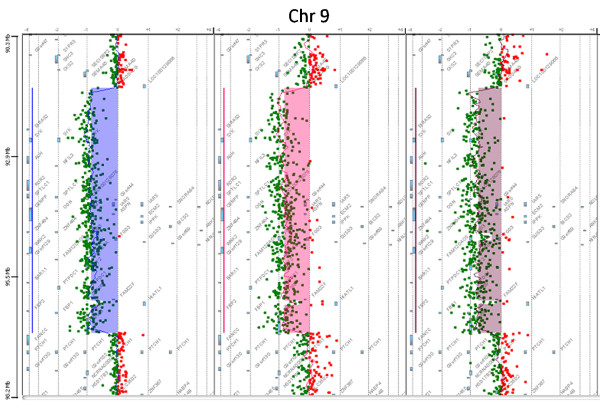
**Array CGH results revealed a deletion at 9q22 in all three patients **Array CGH results of patients 1, 2 and 3 revealed a 5.3 Mb deletion in all patients at 9q22.2q22.32. The shaded area indicates the deleted area with an average log2-ration of -1, indicating loss of one copy of the genomic segment.

Both array CGH and SNP array analysis revealed an identical 5.3 Mb deletion of chromosome 9q22.2q22.32 in all three patients, ranging from basepair 91,523,558 to 96,858,929 (Agilent probes A_16P18688441-A_16_P02135862), covering 30 genes (Figure 4). The average log2-ratio was -1, indicating a one-copy deletion.

The parents of patient 3 and the older sister of patients 1 and 2 were studied for the microdeletion with normal results. Targeted FISH-analysis of at least 80 metaphase spreads, using BAC-probe RP11-30L4, was conducted in an effort to detect mosaicism 9q22 in patient 3. All metaphases showed the deletion of 9q22. Although these results do not exclude germline mosaicism of patient 3, the likelihood is small.

## Discussion

**Figure 5:**
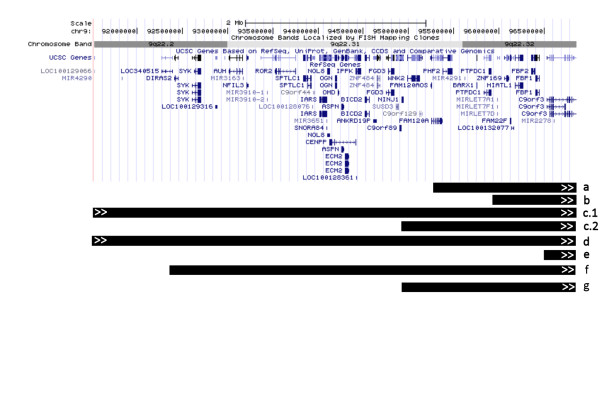
**Overlapping deletions at 9q22 **The figure depicts the genomic segment deleted in the patients of this study, as displayed in the UCSC Genome Browser (http://genome.ucsc.edu). Reported deletions of 9q22 that overlap the deletion found in this study are marked by black bars; a) Redon et al. [7], patients 1 and 2, b) Shimojima et al. [5], c.1) Fujii et al. [16] patient G5, c.2) Fujii et al. [16] patient G10, d) Nowakowska et al. [17], e) de Ravel et al. [14], f) Boonen et al. [15], g) Kosaki et al. [28]. Arrows on the black bar indicate that the deletion starts or ends outside the area shown in the picture.

The clinical and molecular findings of the previously reported 27 live patients and our 3 patients with constitutional 9q22 deletions are summarized in Table 1. Two prenatally terminated fetuses are not included in the analysis [12,13]. Based on available breakpoint information, at least eight reported patients have an overlapping deletion with the one detected in this family (Figure 5) [5,7,14-17].

The deletion found in our three patients leaves the *PTCH1 *gene intact. Instead, according to DECIPHER database (the DECIPHER consortium, http://decipher.sanger.ac.uk/), three genes in the deleted area, viz. *SYK, IARS *and *ASPN*, are scored as likely haploinsufficient [18]. *SYK *is involved in several important biological processes as discussed below. An allelic variant of *ASPN *has been implicated in osteoarthritis [19] and lumbar disc degeneration [20]. Neither of these features was present in our patients. *IARS *encodes an isoleucyl-tRNA synthetase protein and deletions of this gene have not been associated with human disease. In addition, three clustered micro-RNAs (miRNA) called hsa-let-7a, hsa-let-7f and hsa-let-7d are found in the deleted area.. All of these miRNAs have a common primary target gene, *HMGA2*, as scored by Target Scan (http://www.targetscan.org/) and miRBase (http://www.mirbase.org/), indicating that they all function as repressors of this gene. The deletion is flanked by some short segmental duplications. These, however, do not lie at the breakpoints but further up-/downstream and thus it is unclear whether the rearrangement could initially have been due to non-allelic homologous recombination (NAHR).

Almost all patients published so far have been reported to have dysmorphic facial features. However, no unique facial gestalt does emerge. All three patients described here had similar mild dysmorphic features: downward slanting palpebral fissures, high bridged narrow nose with small nares, deep grooved long philtrum, tented and thin upper lip, ears with broad helices and uplifted lobuli. In addition patients 1 and 3 have small toenails.When compared with the previous reports, only the patient reported by Olivieri et al. (2003) has facial features closely resembling those of our patients [8].

A significant common feature in our three patients is incessant middle ear and upper respiratory tract infections. No major immunological deficiency, however, was found to cause the vicious circle of infections, and in the father this symptom disappeared at his teens. Only three previously reported patients suffered from recurrent respiratory infections [14,15,21]. Interestingly, the region deleted in our patients includes the gene *Spleen Tyrosine Kinase *(*SYK*) (MIM *600085) which is widely expressed in hematopoietic cells and other cells of epithelial origin. *SYK *plays an important role in regulation of innate immunity and inflammatory response, and is involved in several human diseases such as allergy, autoimmunity, and haematological malignancies (reviewed by Mócsai et.al [22]). The infections seen in our patients could be linked to reduced *SYK *activity and thereby reduced, albeit not lacking, ability to activate the inflammatory response. Recent studies in mice show that a *SYK*-deletion reduces their antibacterial host defence [23]. *SYK *is deleted in the patients with recurrent respiratory infections reported by Pfeiffer et al., and Boonen et al. but not de Ravel et al. [14,15,21]. The relevance of its haploinsufficiency to human recurrent early-age upper airway infections might be a subject for further studies.

Mental retardation among the reported 9q22 deletion patients is common and usually moderate to severe. Only the 4-year-old patient of Shimojima et al. with a 2,3 Mb deletion is reported to have normal development [5]. The intelligence of the father in our family is within normal limits although a formal neuropsychological testing has not been performed. Instead, his two daughters carrying a similar deletion are moderately retarded. In genetic counseling a similar deletion found both in patients with mental retardation and in a family member with normal cognitive function understandably poses difficulties. The cause of the variable expression remains to be found. According to the Database of Genomic Variants (http://projects.tcag.ca/variation) the deletion described here has not been found in healthy controls. Reduced penetrance was not found to be due to mosaicism admitting the fact that in addition to lymphocytes no other tissue like semen or buccal cells were examined. The parents did not consider further studies for mosaicism important and thus it was not pursued.

Another explanation for variable expression would be two simultaneous genomic hits acting either independently or by affecting the same signaling pathway [24]. In our family the first hit would be the deletion, but the second hit would need other methods to be detected. The paternal microdeletion could also reveal a maternal allelic recessive defect and the consequent autosomal compound heterozygosity in the two daughters would be the cause of their cognitive impairment. No obvious candidate gene is situated in the deleted region. Microdeletions are increasingly frequently encountered associated with the phenomenon of variable penetrance and even nonpenetrance, 15q13.3 microdeletion syndrome being one example [25]. The deleted region is not known to be affected by imprinting that could cause cognitive differences among the patients.

A common developmental problem of our three patients is dysarthria, which is severe and making even the speech of the cognitively normal father difficult to comprehend. A similar problem has not been described in the previously published patients and further studies are needed to explore whether a locus for dysarthria resides in the region defined by the deletion.

Both daughters in our family had symptoms suspected to be epileptic although the diagnosis could not be confirmed. Eight patients from the literature suffer from epilepsy, but no characteristic seizure type emerged [1, 7, 10, 26 (two patients), 16 (two patients), 27].

Patient 3 had unilateral hypoplastic kidney and vesicoureteral reflux. Also three previous patients had a renal problem; hydronephrosis, renal dysplasia or hydroureter [10,16,28]. Interestingly, patient 3 had a congenital chest deformity similar to one of the diagnostic criteria of NBCCS [29]. Additional X-rays could have clarified the presence of possible other NBCCS related skeletal signs. The 9q22 deletion detected did not, however, include *PTCH1 *gene and since our patient otherwise had no indications for further X-ray examinations, it was considered clinically inappropriate to take them. The patient's chest deformity can be independent of the deletion since patients 1 and 2 do not have thoracic anomalies. Yet, one is tempted to speculate that it is a sign that the deletion might effect the expression of *PTCH1*.

Another clinically important gene included in the deletion is *ROR2*. Gain of function mutations of one allele are known to cause brachydactyly type B1 while a mutation of both alleles cause Robinow Syndrome [30,31]. However, haploinsufficiency of *ROR2 *has no effect on phenotype as shown by Oldridge et al., who described two unrelated patients with de novo deletions at 9q22 [30]. Accordingly, none of the six previous patients, nor our patients, whose deletion includes *ROR2*, have either brachydactyly type B1 or features of Robinow Syndrome [[3]3 (restudied in 15), [8,15-17]] (Table 1).

Shimojima et al. suggested that del 9q22 might be a novel overgrowth syndrome by paternal imprinting [5]. Among the previous patients eight carry a deletion in the paternally inherited chromosome [[3] (patient 1), [5,7] (patients 1 and 2), [8,28]] and three are maternally deleted [[3] (patient 2), [9,10]]. These small numbers are naturally liable to bias. Indeed, the paternally deleted patients seem to have a large neonatal size. Among those with data on postnatal growth 3/5 are mildly tall and 4/6 have macrocephaly. Growth data of the three maternally deleted patients is scanty but none of them seems to be tall or to have macrocephaly. The maternal deletions reported appear to be much larger which implies a more significant genomic imbalance and could alone explain differences in phenotype (Table 1).

Recently Kosaki et al. [28] narrowed the region of the proposed overgrowth factor closer to *PTCH1 *gene by their patient's deletion in relation to that of the patients published by Shimojima et al. and Redon et al. [5,7]. Our family's deletion helps to bring it even closer to *PTCH1*. On the other hand tallness and macrocephaly are also inherent features of NBCCS [32]. In a thorough survey of a large series of NBCCS patients Kimonis et al. observed no imprinting effect regarding any feature of the syndrome including macrocephaly [29]. Overgrowth like macrocephaly observed in del 9q22 patients could thus simply reflect the simultaneous haploinsufficiency of *PTCH1*.

Our family underlines the importance to try and include both parental samples in array molecular karyotyping. In a more usual situation with only one developmentally retarded child in the family one might for several reasons be tempted to accept the analysis of the child alone or with only one parent and unknowingly thus get misleading information of the situation. Microdeletion 9q22 adds to the increasing number genomic imbalances that challenge genetic counseling.

## Conclusions

In conclusion, 9q22 deletions are rare and both phenotypically and molecularly unique. In the majority the deletion contains the *PTCH1 *gene, which signifies that the patients in addition to the common mental retardation also develop the tumor proneness syndrome of Gorlin, which is to be taken into account in counseling and follow-up. We present the first familial 9q22 deletion, a father and his two developmentally delayed daughters. Their deletion leaves the *PTCH1 *gene intact. The father does not have significant cognitive problems but has renal and thoracic cage malformations while the daughters do not have congenital malformations. Dysarthric speech and prolonged tendency to ear and upper respiratory infections are common to all three. Major differences in psychomotor development warrant cautiousness in genetic counseling in patients with similar deletions.

## Consent

Written informed consent was obtained from the father, the mother and the paternal grandparents for publication of this case report and accompanying images. A copy of the written consent is available for review by the Editor-in-Chief of this journal.

## Competing interests

The authors declare that they have no competing interests.

## Authors' contributions

LS carried out the molecular genetic studies, participated in the design of the study and drafted the manuscript. MP carried out the clinical studies, participated in the design of the study and drafted the manuscript. MS carried out the sample collection. TM composed the photos for publication. TY and KS performed the FISH analysis and helped to draft the manuscript. JI critically revised the manuscript. SK participated in the study design and coordination and critically revised the manuscript. All authors read and approved the final manuscript.
